# Identification and Expression Analysis of an Interacting Protein (LvFABP) that Mediates *Vibrio parahaemolyticus* AHPND Toxin Action

**DOI:** 10.3389/fimmu.2022.940405

**Published:** 2022-07-04

**Authors:** Xiaoqian Gu, Mei Liu, Baojie Wang, Keyong Jiang, Lei Wang

**Affiliations:** ^1^ Chinese Academy Sciences (CAS) and Shandong Province Key Laboratory of Experimental Marine Biology, Center for Ocean Mega-Science, Institute of Oceanology, Chinese Academy of Sciences, Qingdao, China; ^2^ Laboratory for Marine Biology and Biotechnology, Qingdao National Laboratory for Marine Science and Technology, Qingdao, China; ^3^ University of Chinese Academy of Sciences, Beijing, China; ^4^ Shandong Key Laboratory of Disease Control in Mariculture, Marine Science Research Institute of Shandong Province, Qingdao, China

**Keywords:** acute hepatopancreatic necrosis disease, *Vibrio parahaemolyticus*, *Litopenaeus vannamei*, mechanism, yeast two-hybrid assay, LvFABP, RNA interference, interacting protein

## Abstract

Acute hepatopancreatic necrosis disease (AHPND) caused by *Vibrio parahaemolyticus* causing AHPND (VP_AHPND_) is the most serious disease affecting shrimp farming. The PirA^vp^ and PirB^vp^ toxins of VP_AHPND_ are known virulence factors. However, the corresponding target protein in shrimp that mediates their action has not been identified. By screening yeast two-hybrid cDNA libraries from intestine, stomach, and hepatopancreas of *Litopenaeus vannamei*, the protein with the largest increase in gene expression in shrimp hepatopancreas in response to VP_AHPND_ challenge was identified and designated LvFABP. Analysis revealed high sequence homology of the *LvFABP* gene and a lipocalin/cytosolic fatty acid binding gene. Yeast two-hybrid pairwise analysis, GST-pull down assay, and far-western blot assay were performed to determine the interaction between LvFABP and PirB^vp^. LvFABP was able to directly bind to PirB^vp^. The expression of *LvFABP* in the hepatopancreas was significantly higher at P23 and P27 developmental stages of *L. vannamei*. RNA interference (RNAi) of *LvFABP* reduced the mortality, histopathological signs of AHPND in the hepatopancreas, and the number of virulent VP_AHPND_ bacteria in the intestine, stomach, and hepatopancreas after VP_AHPND_ challenge. We concluded that the LvFABP was involved in AHPND pathogenesis and acted as a VP_AHPND_ toxin interacting protein. This is the first identification of VP_AHPND_ toxin interacting protein from the shrimp digestive system by yeast two-hybrid library screening and were confirmed by *in vitro* protein interaction verification and *in vivo* challenge experiments. This study provides novel insight into the contributions of LvFABP towards AHPND pathogenesis in shrimp. The findings could inform AHPND preventative measures in shrimp farming.

## Introduction

Acute hepatopancreatic necrosis disease (AHPND) in shrimp farming is a bacterial infection that mainly affects *Litopenaeus vannamei* and *Penaeus monodon* (1). The early stage of the disease, which mainly occurs in the seedling stage of shrimp, is termed early mortality syndrome (EMS) (2). Since 2010, the disease has disseminated globally, with direct annual economic losses of more than US one billion dollars to the global shrimp culture industry.

Effective prevention and control of AHPND has become a focus of aquaculture research. The application of antibiotics to effectively control aquaculture diseases has promoted the development of the aquaculture industry (3). However, the growing use of antibiotics increases the likelihood of antibiotic-resistant strains, further accumulation of drug residues, and destruction of aquatic animal microecological balance. These detriments make aquaculture diseases increasingly difficult to control. The quality and safety of aquatic products cannot be guaranteed (4, 5). Disinfectants, water protectants, probiotics, and chinese herbal medicines are used for the prevention and control of AHPND (6–11). However, these methods have limited effects and cannot improve prevention and control of a specific pathogen.

The bacteria that cause AHPND initially colonize the stomach of shrimp. The resulting range of observable symptoms include drowsiness, empty midgut and stomach, and pallor and atrophy of the hepatopancreas (12, 13). Histological analysis of the hepatopancreas has revealed the exfoliation of tubule epithelial cells in the early stages of AHPND and extensive blood cell infiltration in the late stages of infection (14, 15). The fatality rate exceeds 90% within 4 to 5 days (16, 17).

*Vibrio parahaemolyticus* causing AHPND (VP_AHPND_) harboring the pVA1 plasmid is the main pathogenic microorganism. The pVA1 plasmid contains gene sequences encoding two photorhabdus insect-related (Pir) toxins, PirA^vp^ and PirB^vp^, which are the main factors leading to intestinal and hepatopancreas injury in shrimp (18). PirB*^vp^* alone has the ability to cause cell damage (13, 18). However, full toxicity is caused when both PirA*^vp^* and PirB*^vp^* are present. The *PirA^vp^* and *PirB^vp^* genes are part of the same operon (18). The genes are synchronously regulated and expressed (19). However, how VP_AHPND_ colonizes the stomach, hepatopancreas, and intestinal tract, and how the PirA^vp^ and PirB^vp^ toxins interact with these tissues are unknown. Clarifying the mechanisms of these interactions in *L. vannamei* is crucial for the development of disease prevention and control strategies and agents, and to reduce the mortality and economic in shrimp aquaculture.

Structural analyses have revealed the functional relationship between PirA^vp^/PirB^vp^ and the Cry toxin of *Bacillus thuringiensis* (19). The Cry pore-forming toxin includes three functional domains: N-terminal pore-forming domain I, middle receptor binding domain II, and C-terminal sugar/receptor binding domain III (19). The toxic mechanism of Cry toxins involves the recognition of n-acetylgalactosamine, which is present on several receptors, by domain III. Domain II then binds to the recognized receptor. Finally, the α1 helix of domain I is hydrolyzed to form pores on the membrane (20–24). Given their structural similarities, the cytotoxic activation of PirA^vp^ and PirB^vp^ should be similar to that of the Cry toxins. If so, finding the receptor associated with PirA^vp^ and PirB^vp^ is crucial for the treatment of AHPND disease.

In the present study, we identified *L. vannamei* fatty acid binding protein (LvFABP), a potential target protein of VP_AHPND_ toxin, by screening a yeast two-hybrid cDNA library of VP_AHPND_ challenged *L. vannamei*. To investigate the function of LvFABP as a toxin interacting protein, yeast two-hybrid pairwise analysis, GST-pull down assay, and far-western blot assay of protein–protein interactions between LvFABP and PirB^vp^ toxin were performed to confirm the LvFABP–toxin interaction. The gene expression pattern of LvFABP in different developmental stages and tissues, and the survival of *L. vannamei* challenged by VP_AHPND_ after RNAi of *LvFABP* was conducted to further confirm the role of protein LvFABP in disease occurrence. These studies will deepen the understanding of the internal mechanism of LvFABP involved in disease occurrence and could provide new strategies for treatment of AHPND.

## Materials and Methods

### Experimental Animals, Bacterial Strains, and Ethical Statement

The experimental shrimp in the post-larvae stage were cultured in the aquarium of the Institute of Oceanology, Chinese Academy of Sciences (Qingdao, Shandong, China). *V. parahaemolyticus* VP-E1 strain was donated by the Yellow Sea Fisheries Research Institute, Chinese Academy of Fishery Sciences. All animal experiments were performed in accordance with accepted standards of humane animal care. No endangered or protected species were used.

### Screening and Sequence Analysis of Putative VP_AHPND_ Toxin Interacting Protein

A yeast two-hybrid assay was performed to identify PirB^vp^-interacting proteins. A *L. vannamei* cDNA library was constructed by Personalbio Biotech Company (Shanghai, China). The Y2H gold yeast transporter containing the correct pGBKT7-PirB^vp^ bait plasmid was used as the receptor to prepare the competent cells. The pGADT7-shrimp were transformed with the cDNA library plasmids using SD/-Leu/-Trp/-His/-Ade medium. Polymerase chain reaction (PCR) and DNA sequencing were performed for yeast positive clone colonies. Finally, BLAST analysis was performed for sequences in GenBank database.

A BLAST search (http://blast.ncbi.nlm.nih.gov/Blast.cgi) was used to identify the LvFABP sequence. The DNAMAN software package (http://www.lynnon.com/) was used for multiple sequence alignment. The motifs were analyzed using Motif Search (http://www.genome.jp/tools/motif/). Transmembrane helices of LvFABP amino acids were predicted by the TMHMM 2.0 Server (http://www.cbs.dtu.dk/services/TMHMM/).

### Cloning of Full-Length cDNA of the *LvFABP* Fatty Acid Binding Protein Gene of *L. vannamei*

The LvFABP1-F/R primer pairs (**Table 1**) were designed based on the full-length CDS sequence of *P. monodon* gene published by GenBank (DQ459988) to amplify the gene fragment of *LvFABP* in *L. vannamei*. The PCR amplification product was purified and recovered according to the instructions of the Agarose gel DNA recovery kit (Transgen Biotech, Beijing, China). The PCR specific amplification product was then connected to the PGEM-Teasy vector (Tsingke Biotech, Beijing, China). The recombinant plasmid was transformed into *Escherichia coli* Top10. The obtained transformants were incubated on LB medium (50 μg/ml kanamycin) with constant shaking at 150 rpm at 37°C. Positive clones were selected and verified by the bacterial solution PCR DNA sequencing.

**Table 1:** The primers of candidate genes used for real-time quantitative PCR (qRT-PCR), RNAi and gene cloning.

Gene name	Primer sequence (5′-3′)
pirA^Vp^-F	TTGGACTGTCGAACCAAACG
pirA^Vp^-R	GACCCCATTGGTATTGAATG
pirB^Vp^-F	CTACTTTTCTGTACCAAATTCATC
pirB^Vp^-R	ATGACTAACGAATACGTTGTAAC
LvFABP1-F	ATGAAGGCTCTGGG-TGTTG
LvFABP1-R	CCGTAGTCCCAGTCAT-ATCC
LvFABP-5' RACE	GCAGACAACGTCATCAACCTTGCACTC
LvFABP-3' RACE	GACGGCGATACCTACACAATGAAGACG
pirB^Vp^-F1	CTAGGATCCCTTTTCTGTACCAAATTCATC
pirB^Vp^-R1	ATGCTCGAGACTAACGAATACGTTGTAAC
LvFABP2-F	ATGGGATCCAAGGCTCTGGGTGTTG
LvFABP2-R	CCGCTCGAGTAGTCCCAGTCATATCC
LvFABP3-F	GGCAGGAGCGTCAGTTGT
LvFABP3-R	GTCGCAGCGTTACCCATC
dsEGFP-F	TAATACGACTCACTATAGGGCAGTGCTTCAGCCGCTACCC
dsEGFP-R	TAATACGACTCACTATAGGGAGTTCACCTTGATGCCGTTCTT
dsLvFABP-F	TAATACGACTCACTATAGGGGAGTTGCCCTCGCTGTTTGCTAT
dsLvFABP-R	TAATACGACTCACTATAGGGCAGAAGATGTTACAAGACTAAAG
β-actin-F	GCCCATCTACGAGGGATA
β-actin-R	GGTGGTCGTGAAGGTGTAA

The restriction enzyme sites were underlined.

Primers LvFABP-5’ RACE and LvFABP-3’ RACE needed for 5’ and 3’ RACE, respectively, were designed using the obtained fatty-binding protein gene fragments as templates. LvFABP-5 ‘RACE and LvFABP-3’ RACE primers were used with SMART™ RACE cDNA Amplification Kit (TaKaRa Bio, Shiga, Japan) to synthesize the first strand of cDNA as template. Amplification of 5’ -end and 3’ -end FABP gene sequences was performed according to the reaction system and reaction conditions recommended by SMART™ RACE cDNA Amplification Kit.

### Identification of *V. parahaemolyticus* Causing AHPND (VP-E1)

Strain VP-E1 was cultured in tryptic soy broth (TSB) supplemented with 1.5% NaCl incubated at 30˚C with shaking for 24 h and the DNA was obtained using the gene group extraction reagent box (Transgen Biotech). Primers were designed and PCR was performed to amplify PirA^vp^ and PirB^vp^ full-length sequences of virulence gene based on the sequence of published plasmid pVA1. The PirA^vp^-F/R and PirB^vp^-F/R primers were synthesized by Tsingke Biotechnology Co., LTD (Qingdao, China). The primers are presented in **Table 1**. After the PCR amplification products were purified by the DNA purification kit, the PirA^vp^ and PirB^vp^ target fragments were respectively connected to T clone vectors overnight at 16°C. The connected vectors were transformed into *E. coli* Top10. Positive clones were incubated on LB medium (50 μg/ml kanamycin) with constant shaking at 150 rpm at 37°C and the plasmids were extracted and sequenced by Tsingke Biotechnology Co., LTD.

### Recombinant Expression and Purification of PirB^vp^ and LvFABP

The *PirB^vp^* gene and pET-30(a) were digested by *Bam*HI and *Xho*I endonucleases respectively, then ligated by T4 DNA ligase to construct the recombinant plasmid PirB^vp^+pET-30(a). The *LvFABP* gene and pEGX-4T-1 were digested by *Bam*HI and *Xho*I endonucleases, respectively, then ligated by T4 DNA ligase to construct the recombinant plasmid LvFABP+pEGX-4T-1. The PirB^vp^-F1/R1 and LvFABP2-F/R specific primers were synthesized by Tsingke Biotechnology Co., LTD. The primers are shown in **Table 1**. The recombinant PirB^vp^ and LvFABP were expressed in *E. coli* BL21 (DE3) to obtain the transformants by incubation on LB medium (50 μg/mL kanamycin and 50 μg/mL ampicillin, respectively) with constant shaking at 150 rpm at 37°C. Isopropyl-β-D-thiogalactopyranoside (IPTG) was added (0.1 mM) to induce the protein when the optical density at 600 nm (OD_600_) reached 0.5. Induction occurred during incubation for 16 h at 16°C.

The Ni-NTA His Tag Kit (Novagen, Merck, Darmstadt, Germany) was used to purify the recombinant PirB^vp^. The recombinant PirB^vp^ was washed with binding buffer and eluted with elution buffer with different concentrations of imidazole (20, 40, 80, 120, 160, and 200 mM) (24). Finally, PirB^vp^ was assessed by sodium dodecyl sulfate–polyacrylamide gel electrophoresis (SDS–PAGE) (25).

GST-tag Protein Purification Kit (Beyotime, Shanghai, China) was used to purify the recombinant LvFABP. Cracking buffer solution was used to wash the miscellaneous protein. Elution buffer (50 mM Tris, 150 mM NaCl, 10 mM GSH, pH 8.0) was used to elute the recombinant LvFABP. Finally, the target protein LvFABP was assessed by SDS–PAGE.

### Yeast Two-Hybrid Pairwise Analysis of Interaction Between LvFABP and PirB^vp^

To detect the pairwise protein–protein interaction between LvFABP and PirB^vp^, pGBKT7 bait vector containing full-length cDNA of PirB^vp^ together with the pGADT7 prey vector containing full-length cDNA of LvFABP were co-transformed into AH109 yeast cells. The transformants were plated on SD/-Leu/-Trp medium and the positive clones were then grown on SD/-Leu/-Trp/-His/-Ade medium to recognize the protein–protein interaction. pGADT7-largeT/pGBKT7-p53 was used as a positive control, and pGADT7-largeT/pGBKT7-laminC was used as a negative control.

### GST-Pull Down and Far-Western Blot Assays of Interaction Between LvFABP and PirB^vp^

To investigate the interaction between the PirB^vp^ toxin and LvFABP, the PirB^vp^-His, LvFABP-GST, and GST genes were cloned and heterologously expressed in *E. coli* BL21. The purified recombinant PirB^vp^-His and LvFABP-GST protein had a single band on SDS–PAGE. The GST Protein Interaction Pull-down Kit (Thermo Fisher Scientific, Waltham, MA, USA) was used to verify the interaction between LvFABP and PirB^vp^. Immobilized glutathione was completely resuspended, 100 μL aliquots of 50% immobilized glutathione were added to four centrifuge tubes and centrifuged at 1200 × g for 2 min. The supernatant was discarded and the pellet was resuspended in 500 μL PBS and centrifuged at 1200 × g for 2 min. The supernatant was discarded. The PBS wash was repeated three times. LvFABP-GST and PirB^vp^-His proteins, GST and PirB^vp^-His proteins, and PBS and PirB^vp^-His proteins were added to glutathione resin and the mixture was rotated during an overnight incubation at 4°C. The sample was then centrifuged at 1200 × g for 5 min and the supernatant was completely removed. The pellet was resuspended in 500 μL PBS, centrifuged at 1200 × g for 2 min, and the supernatant was removed. This washing was repeated three times. After adding 50 μL elution buffer, the solution was rotated during incubation at 4°C for 20 min. The sample was centrifuged at 1200 × g for 2 min and the supernatant was discarded. The centrifuged liquid was placed on ice and 20 µL aliquots were analyzed by SDS–PAGE. The resolved proteins were transferred to a nitrocellulose membrane. The membrane was incubated with sealed by bovine serum albumin (BSA) to block non-specific binding sites. PirB^vp^ was co-incubated with the treated membrane to bind PirB^vp^ to the interacting protein immobilized on the membrane. Unbound decoy protein was washed off after incubation. After incubation with primary antibody (1:2000) and secondary antibody (1:5000) of PirB^vp^ toxin, chemiluminescence was used to detect the presence of target protein (PirB^vp^ toxin). The secondary antibodies were constructed by Genecreate Biotech Company (Wuhan, China).

The far-western blot assay was also used to verify the interaction between LvFABP and PirB^vp^. LvFABP was separated by SDS–PAGE and transferred to a nitrocellulose membrane. After transfer, the membrane was treated with BSA as described above. The bait protein (PirB^vp^ toxin) was co-incubated with the blocked membrane to bind PirB^vp^ to the immobilized interacting protein. As above, the unbound decoy protein was washed off after incubation. After incubation with the primary antibody (1:2000) and secondary antibody (1:5000) of PirB^vp^ toxin, chemiluminescence was used to detect the presence of target protein.

### Expression of *LvFABP* in Different Tissues of *L. vannamei* at Different Periods

Eighty healthy shrimps with similar body condition were selected under the same feeding conditions. Hepatopancreas were collected at different post-larval stages (P23, P27, P30, P34, P37, P41, P44, and P47), with 10 shrimps at each time point. The shrimp were preserved by immediate immersion in liquid nitrogen. Intestine, stomach, and hepatopancreas tissues (2~5 g each) were collected quickly from shrimps in the various post-larval stage, and immediately preserved in liquid nitrogen to analyze the expression of *LvFABP* in *L. vannamei*. RNA extraction and cDNA reverse synthesis were performed. cDNA was used as the template for fluorescence quantitative PCR. The TransStart Top Green qPCR SuperMix kit (Transgen Biotech) was used for real-time quantitative PCR, with *β-actin* as internal reference. The primers used were LvFABP3-F/R and β-actin-F/R (**Table 1**).

### Tissue Distribution Analysis of *LvFABP* mRNA After VP_AHPND_ Challenge

Shrimp tissues were collected 48 h after infection with VP_AHPND_ strain VP-E1 to analyze the *LvFABP* expression in different tissues (intestine, stomach, and hepatopancreas) of *L. vannamei*. RNA extraction and cDNA reverse synthesis were performed. cDNA was used as the template for fluorescence quantitative PCR. The PCR conditions and primers were the same as described just above.

### *In vivo* RNAi

To investigate the functional importance of LvFABP as a putative VP_AHPND_ toxin interacting protein, RNAi was used to silence the expression of *LvFABP* by the injection of double-stranded RNA (dsRNA). The 300 bp ds*LvFABP* was designed according to the conservative domain of LvFABP. Primers dsLvFABP-F and dsLvFABP-R were designed to amplify ds*LvFABP* and primers dsEGFP-F and dsEGFP-R were used to amplify control ds*EGFP*. ds*LvFABP* was synthesized *in vitro* using the TranscriptAid T7 High Yield transcription kit (Thermo Fisher Scientific). The primers used are shown in **Table 1**.

To optimize the silencing efficiency of ds*LvFABP*, different RNAi doses were used and ds*LvFABP* was diluted with 0.85% NaCl to different concentrations. The final RNAi/ds*LvFABP* conditions were 2 µg/10 µL, 6 µg/10 µL, and 10 µg/10 µL. One experimental group (ds*LvFABP*) and two control groups (NaCl and ds*EGFP*) were set and dsRNA was injected into the third muscle side of the tail of *L. vannamei*. Three biological replicates were performed. After 48 h of interference, three shrimp in each group were collected as a sampling unit. RNAi efficiency of each dsRNA dosage was determined. This led to the use of the 10 μg/10 μL condition for subsequent injections into shrimp in RNAi experiments. One experimental group (ds*LvFABP*) and two control groups (NaCl and ds*EGFP*) were established. dsRNA was injected once every 4 days into the third muscle side of the tail of *L. vannamei*. Three biological replicates were performed. The RNAi experiment lasted for 2 weeks. Water was changed daily. The shrimp were fed the same amount of food in the morning, mid-day, and at night. After the 2-week interference, three shrimp in each group were collected as a sampling unit. Total RNA was isolated and a RevertAid First Strand cDNA Synthesis Kit (Thermo Fisher Scientific) was used to synthesize the cDNA. The gene expression level of *LvFABP* was analyzed by the qRT-PCR to verify the efficiency of dsRNA silencing.

### Survival Rate of VP_AHPND_ Challenged *L. vannamei* after *LvFABP* Silencing

The ds*LvFABP*, ds*EGFP*, and NaCl groups (n=10 *L. vannamei* per group) were established. dsRNA was injected once every 4 days in the third muscle side of the tail of each *L. vannamei*. Three biological replicates were performed. The experiment period was 2 weeks, with fresh water daily and feeding of the same amount of food in in the morning, mid-day, and night. Two weeks later, the treated shrimp were challenged with VP-E1 **(**10^7^ CFU/mL). In the corresponding three control groups (10 shrimp per group), the shrimp were challenged with PBS (pH 7.4) instead of the AHPND-causing *V. parahaemolyticus*. After 7 days of challenge, the survival rate of shrimp was observed at 0, 12, 24, 48, 84, 168 h post-infection. Gene silencing efficiency is essential for the survival of infection, while gene silencing efficiency fluctuates. Thus we also studied the silencing efficiency at different times of *LvFABP*. qRT-PCR was used to measure the mRNA expression levels of target genes after different periods of interference.

### Effect of *LvFABP* Silencing on Intestine, Stomach, and Hepatopancreas Colonization by VP-E1

After *LvFABP* silencing, shrimp intestine, stomach, and hepatopancreas tissues were collected before (unchallenged) and after challenge with VP-E1 (10^7^ CFU/mL). The *pirA^vp^* copy number in the intestine, stomach, and hepatopancreas tissues of *L. vannamei* was detected by qRT-PCR to measure the colonization of the pathogenic *V. parahaemolyticus*. The previously described regression equation of the qPCR standard curve (26) for quantitative detection of *pirA^vp^* copy number in the intestine, stomach, and hepatopancreas tissues of *L. vannamei* was:


Ct=−3.235lg(N)+37.555

where N is the copy number and Ct is the cycle number. The copy number was calculated according to the Ct value of the unknown sample. TransStart^®^ Top Green qPCR SuperMix (Transgen Biotech) was used for qRT-PCR. Ct values of samples were obtained to detect *pirA^vp^* copy number in the tissues of *L. vannamei* after LvFABP silencing.

### Antibacterial Effect of LvFABP Protein on VP_AHPND_ Strain VP-E1

VP-E1 was inoculated in tubes containing TSB supplemented with 1.5% NaCl incubated at 30°C with shaking for 24 h. The resulting cultures were each diluted in PBS to produce suspensions containing 10^6^ CFU/mL of bacteria. LvFABP protein solution of 10 mg/mL was undiluted or double-diluted to produce 5, 2.5, 1.25, 0.625, 0.3125, 0.1625, and 0.0812 mg/mL solutions. Two hundred microliter aliquots of each VP-E1 suspension were added towels of a 96-well plate, followed by addition of 100 μL of the different concentrations of LvFABP protein. The control wells received 100 μL PBS. Three experiments were repeated in each group. After incubation at 30°C for 24 h, the turbidity was recorded by a microplate reader.

## Results

### Characterization and Sequence Analysis of LvFABP

**Figure 1:**
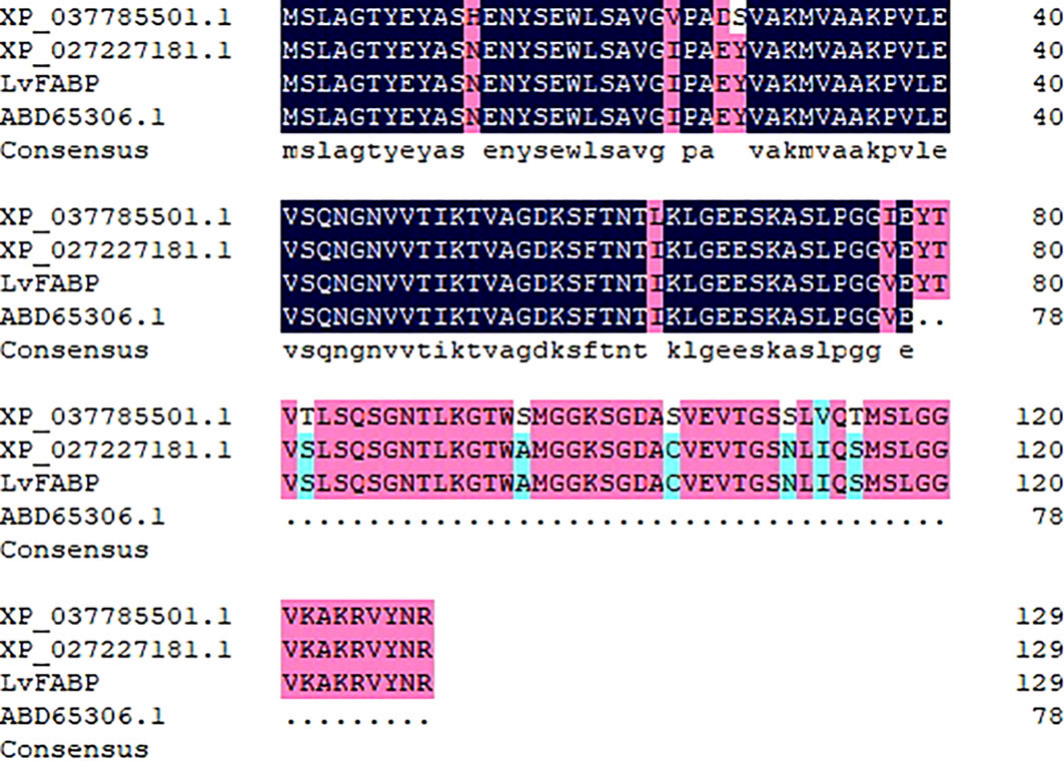
Multiple sequences alignment of LvFABP. The black columns indicated completely identical amino acid zones, the red columns indicated ≧75% identical amino acid zones, the blue columns indicated ≧50% identical amino acid zones. The percent identity between LvFABP and the fatty acid binding protein 10 isolated from *Penaeus vannamei* (ABD65306.1) was 78%; The percent identity between LvFABP and the fatty acid binding protein 1-B.1-like isolated from *Penaeus monodon* (XP_037785501.1) was 90%; The percent identity between LvFABP and the fatty acid binding protein 1-B.1-like isolated from *Penaeus vannamei* (XP_037785501.1) was 100%.

Screening yeast two-hybrid cDNA libraries from intestine, stomach, and hepatopancreas tissues of *L. vannamei* identified a total of nine proteins that interacted with PirB^vp^. The proteins with the largest increase in expression was found in hepatopancreas tissues after shrimp were challenged with VP_AHPND_. This protein displayed extensive sequence homology with a lipocalin/cytosolic fatty acid binding protein. Thus, the protein was designated LvFABP. The full coding sequence of the *LvFABP* gene was 1,042 bp, with a 411 bp open reading frame. The sequence encodes a protein composed of 136 amino acids with a theoretical molecular weight of 13.63 kDa. There are 113 bp (5’ end) and 518 bp (3’ end) untranslated regions on the respective ends of the gene. Sequence analysis showed that the encoded protein belongs to the lipocalin/cytosolic fatty acid binding protein family. The LvFABP protein sequences were compared with those of the reported proteins ABD65306.1, XP_037785501.1 and XP_027227181.1 (**Figure 1**). The deduced amino acid sequence of LvFABP was highly similar to these genes (78%–100%).

### Yeast Two-Hybrid Pairwise Analysis, GST-Pull Down Assay, and Far-Western Blot Assay of Interaction Between LvFABP and PirB^vp^

**Figure 2:**
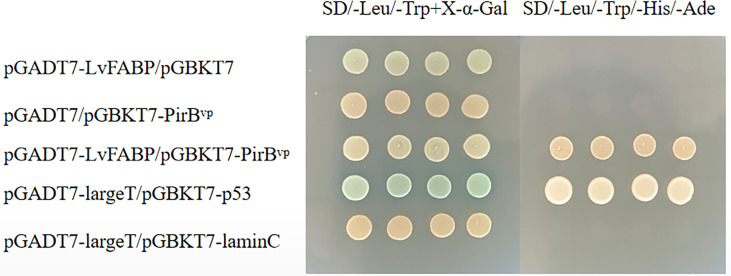
Yeast two-hybrid pairwise analysis of the interaction between LvFABP and PirB^vp.^ There were five groups of experiments: pGADT7-LvFABP/pGBKT7, pGADT7/pGBKT7-PirB^vp^, pGADT7-LvFABP/pGBKT7-PirB^vp^, pGADT7-largeT/pGBKT7-p53, and pGADT7-largeT/pGBKT7-laminC. pGADT7-largeT/pGBKT7-p53 was used as a positive control, and pGADT7-largeT/pGBKT7-laminC was used as a negative control.

The yeast two-hybrid pairwise analysis results showed that all the co-transferred BD and AD vector AH109 strains were cloned on medium lacking SD/-Leu/-Trp, indicating that the co-transformation was successful (**Figure 2**). The clones on SD/-Leu/-Trp medium were selected and cultured in SD/-Leu/-Trp/-His/-Ade plates. AH109 of the experimental group and the co-transformation positive control plasmid grew normally out of the clones, while clones were not detected in the negative control group (**Figure 2**). These findings indicated the presence of an interaction between LvFABP and PirB^vp^. GST-pull down and far-western blot assays proved the interaction between LvFABP and PirB^vp^. After the successful expression of the recombinant truncated LvFABP-GST, PirB^vp^-His, and GST proteins in *E. coli* BL21, the purified LvFABP bound directly to PirB^vp^ (**Supplementary Figure 1**). Far-western blot assay results also demonstrated binding of purified LvFABP to PirB^vp^ (**Supplementary Figure 2**).

### Expression of *LvFABP* in Different Tissues of *L. vannamei* and at Different Periods

**Figure 3:**
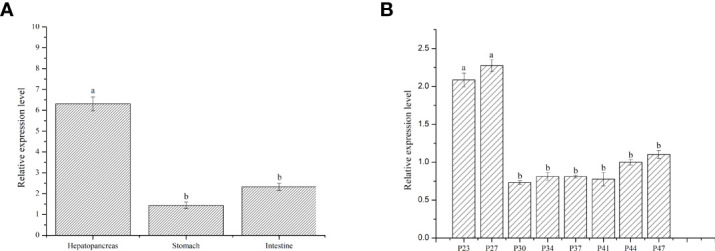
Expression of *LvFABP* in different tissues and hepatopancreas at different periods of *L. vannamei*. **(A)**: the expression of LvFABP in different tissues of *L. vannamei*, the intestine, stomach, and hepatopancreas were collected to analyze; **(B)**: the hepatopancreas at different periods of *L. vannamei* were collected to analyze the expression of LvFABP at different developmental stages. Each bar represents the mean ± standard deviation (SD) of triplicate experiments. Different letters indicated significantly difference (P < 0.05).

To study the expression of *LvFABP* in different tissues of *L. vannamei*, the intestine, stomach, and hepatopancreas of post-larval stage shrimps were analyzed. Expression of *LvFABP* in the hepatopancreas was significantly higher than that in other tissues (**Figure 3A**). Hepatopancreas tissue was collected at different developmental stages of *L. vannamei* to analyze the expression of *LvFABP*. *LvFABP* expression at P23, P27, P44, and P47 developmental stages of *L. vannamei* was higher than at P30, P34, P37, and P41. Expression of *LvFABP* at P23, P27 was the most significant in comparison with other periods (**Figure 3B**).

### *LvFABP* Is Upregulated in Response to AHPND

**Figure 4:**
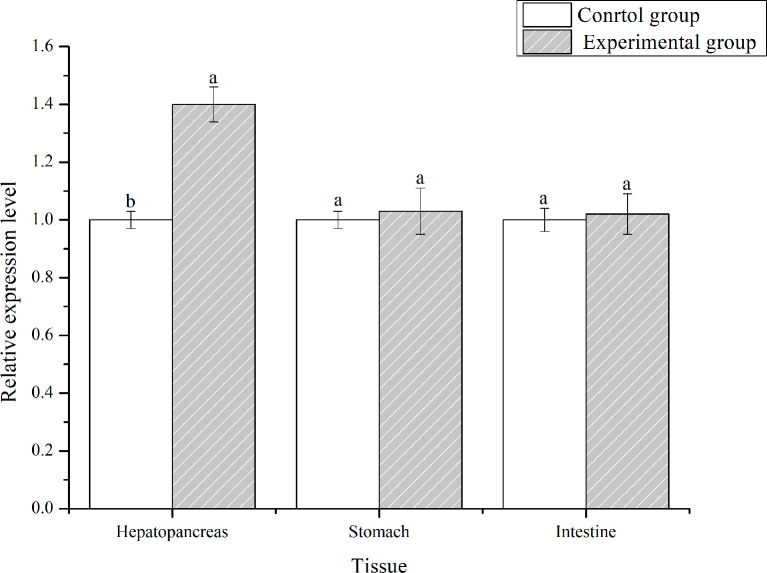
Tissues distribution analysis of *LvFABP* mRNA after VP_AHPND_ challenge. Shrimp tissues (intestine, stomach, and hepatopancreas) were collected after infection with the VP_AHPND_ strain VP-E1 at 48 h. Each bar represents the mean ± standard deviation (SD) of triplicate experiments. Different letters indicated significantly difference (P < 0.05).

PirA^vp^ and PirB^vp^ were determined by sequence alignment, which confirmed the strain VP-E1 was *V. parahaemolyticus* causing AHPND. *L. vannamei* was challenged with the VP_AHPND_ strain VP-E1 to analyze the expression profiles of *LvFABP* in the intestine, stomach, and hepatopancreas using qRT-PCR. The expression of *LvFABP* in the hepatopancreas was significantly upregulated after challenge with VP-E1 (**Figure 4**).

### RNAi of *LvFABP* Increases Survival Rate of Shrimp Infected With VP_AHPND_

**Figure 5:**
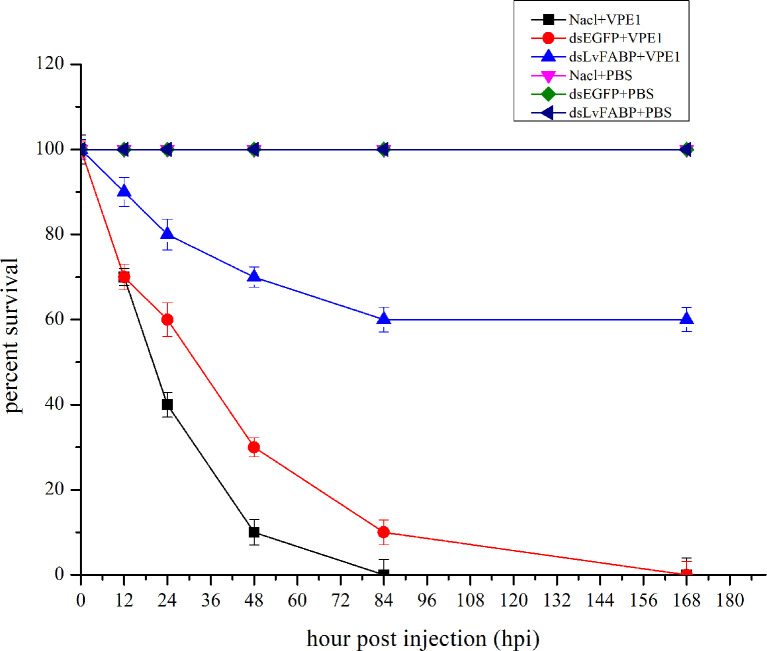
*LvFABP* silencing reduced mortality in shrimp challenged with the AHPND-causing *V. parahaemolyticus* VP-E1 strain. Cumulative mortality was monitored in 3 groups of 10 shrimp for each of the individual experimental conditions. NaCl-injected shrimp were challenged with VP-E1, dsEGFP-injected shrimp were challenged with VP-E1, dsLvFABP-injected shrimp were challenged with VP-E1, NaCl-injected shrimp were challenged with PBS, dsEGFP-injected shrimp were challenged with PBS, dsLvFABP-injected shrimp were challenged with PBS. Shrimp survival was observed every 12 h post treatment for 7 days. All experiments were performed in triplicate and the survival percentage calculated as mean ± standard deviation (SD) at each time point as shown.

**Figure 6:**
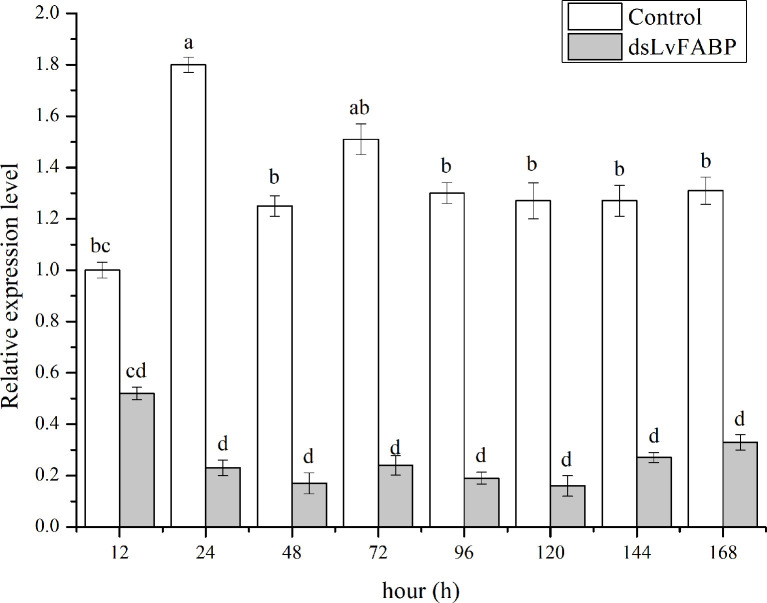
The silencing efficiency at different times of *LvFABP*. The ds*LvFABP*, ds*EGFP*, and NaCl groups (n=10 *L. vannamei* per group) were established. The experiment period was 2 weeks and dsRNA was injected once every 4 days. Two weeks later, the treated shrimps were tested at 12, 24, 48, 72, 96, 120, 144 and 168 h. Each bar represents the mean ± standard deviation (SD) of triplicate experiments. Different letters indicated significantly difference (P < 0.05).

**Figure 7:**
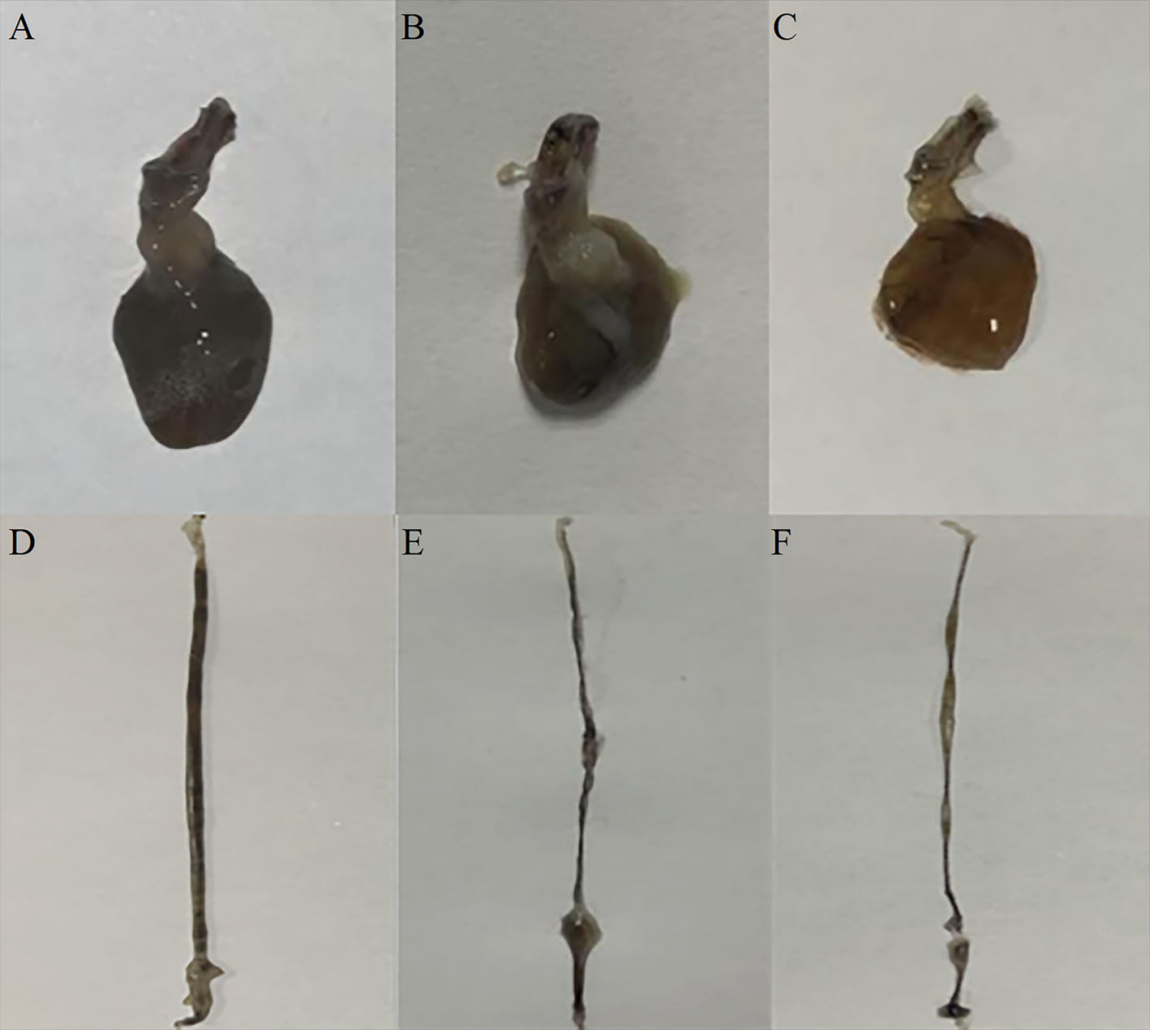
The effect of silencing *LvFABP* expression by dsLvFABP interference on hepatopancreas and intestine morphology of *L. vannamei* challenged with the VP_AHPND_ strain VP-E1. **(A, D)**: hepatopancreas and intestine morphology of *L. vannamei* after dsLvFABP-injected shrimp were challenged with the AHPND-causing *V. parahaemolyticus* VP-E1 strain; **(B, E)**: hepatopancreas and intestine morphology of *L. vannamei* after NaCl-injected shrimp were challenged with the AHPND-causing *V. parahaemolyticus* VP-E1 strain; **(C, F)**: hepatopancreas and intestine morphology of *L. vannamei* after dsEGFP-injected shrimp were challenged with the AHPND-causing *V. parahaemolyticus* VP-E1 strain.

To investigate the function of LvFABP during VP_AHPND_ infection, the expression of *LvFABP* was silenced by injection of double-stranded RNA (dsRNA). The efficiency of the RNAi treatment was measured for each dsRNA dosage. The condition of 10 μg/10 µL was the optimal interference dose (**Supplementary Figure 3**). This was used for injections in shrimp in subsequent RNAi experiments. *LvFABP* expression was significantly reduced in the ds*LvFABP* group compared with the control group injected with ds*EGFP* and NaCl (**Supplementary Figure 4**). When the effect of *LvFABP* silencing on the survival rate of *L. vannamei* challenged with the VP_AHPND_ strain VP-E1 was determined, it was found that *LvFABP* silencing significantly increased the survival rate of *L. vannamei* after VP_AHPND_ infection. The survival rate of *L. vannamei* challenged with VP-E1 after *LvFABP* silencing still exceeded 60% for 168 h (**Figure 5**). The silencing efficiency at different times of *LvFABP* results showed that although it decreased at 12 h after dsRNA injection, it was not statistically significant compared with the control group. At 24 h after ds*LvFABP* injection, the mRNA expression level decreased significantly, and the mRNA level was only 12.8% of that of the control group. The interference efficiency of *LvFABP* was still above 70% at 168 h (**Figure 6**). Analysis of the effect of *LvFABP* silencing on the VP_AHPND_ *pirA^vp^* colonization in the intestine, stomach, and hepatopancreas of *L. vannamei* revealed that the number of *pirA^vp^* in the ds*LvFABP* silenced group was significantly lower than that in the ds*EGFP* and NaCl control groups. After *LvFABP* silencing, *pirA^vp^* copies in the intestine, stomach, and hepatopancreas tissues of *L. vannamei* were significantly decreased, further indicating that injection of ds*LvFABP* could effectively reduce the number of colonizing VP_AHPND_ in *L. vannamei* (**Supplementary Figure 5**). The effect of silencing *LvFABP* expression by ds*LvFABP* interference on hepatopancreas and intestine morphology of *L. vannamei* challenged with the VP_AHPND_ strain VP-E1 was studied. Compared with the ds*EGFP* and NaCl control groups, the general clinical hepatopancreas and intestine symptoms were significantly reduced in the ds*LvFABP* silenced group (**Figure 7**). These collective findings indicate the lack of an effect of LvFABP on VP_AHPND_ infection.

### Antibacterial Effect of LvFABP Protein on VP-E1

**Figure 8:**
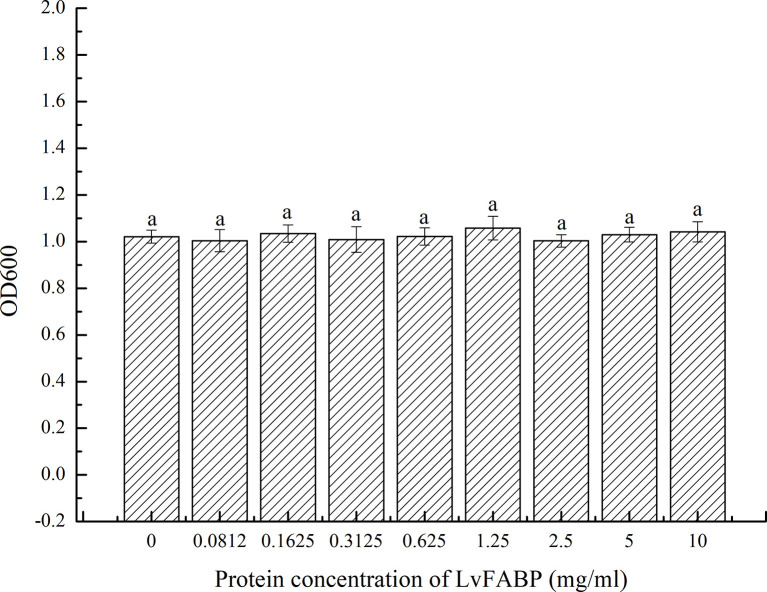
Antibacterial effect of LvFABP protein on VP-E1. VP-E1 suspension (200 μl) prepared was added to the 96-well plate, then LvFABP protein (100 μl) with different concentrations (10 mg/mL, 5 mg/mL, 2.5 mg/mL, 1.25 mg/mL, 0.625 mg/mL, 0.3125 mg/mL, 0.1625 mg/mL, 0.0812 mg/mL) were added, and 100 μl PBS was added to the control group, and three experiments were repeated in each group. After incubation at 30°C for 24 h, the turbidity was recorded by a microplate reader. Each bar represents the mean ± standard deviation (SD) of triplicate experiments.

Examination of LvFABP protein inhibition by VP_AHPND_ strain VP-E1 revealed no antibacterial effect of 10, 5, 2.5, 1.25, 0.625, 0.3125, 0.1625, and 0.0812 mg/mL suspensions of LvFABP protein on VP_AHPND_ strain VP-E1 compared to the control group (**Figure 8**).

## Discussion

AHPND is the most serious disease affecting shrimp farming worldwide. Prevention and control measures of AHPND mainly include strict seedling disease detection, reducing culture density, regulating water quality, strengthening bait feeding management, adding probiotics, and other comprehensive prevention and control measures. However, these actions have not proven effective in the prevention and control problems of AHPND. Findings concerning the Cry protein of *B. thuringiensis* (27) indicate that the toxin receptor protein in the host is an important factor affecting the pathogenicity of toxin protein. Thus, identification of the receptor protein is expected to be a breakthrough in the prevention and control of AHPND.

In this study, LvFABP interacting with PirB was screened from yeast two-hybrid cDNA libraries from *L. vannamei*. The interaction between LvFABP and PirB was confirmed by yeast two-hybrid pairwise analysis and GST-pull down and far-western blot assays. The role of LvFABP in AHPND infection was further studied after the expression of *LvFABP* was suppressed by RNAi. To our knowledge, this is the first toxin interacting protein identified from the shrimp digestive system. The in-depth examinations of its interaction with AHPND toxin are also novel. A previous report described the involvement of LvAPN1 of hemocytes in AHPND pathogenesis as a VP_AHPND_ toxin receptor mediating toxin penetration into hemocytes (28). Since the digestive system is considered the first barrier to VP_AHPND_ infection and since the hepatopancreas is the main target organ with the most obvious symptoms, we believe that the identification of toxin interacting proteins in the digestive system, especially with the hepatopancreas, is significant for disease control. Another report demonstrated that alpha amylase-like protein, a 1,4-α-d-glucan glucanohydrolase targeted by the Cry toxin, interacts with PirB^vp^ (29). Still another recent report used anion-exchange chromatography to reveal the recognition of mucin-like O-glycosidic structures and beta-hexosaminidases by the PirB^vp^ subunit on the membrane of epithelial cells of the hepatopancreas (30). These previous results were based solely on far-western assay, mass spectrometry, or anion-exchange chromatography. Interactions validation between proteins and AHPND toxins *in vitro* and functional validation of the above proteins in mediating VP_AHPND_ infection *in vivo* were not validated.

Sequence analysis showed that protein LvFABP belongs to the lipocalin/cytosolic FABP family. FABPs are members of a multi-gene family of intracellular FABPs, which are widely present in vertebrate and invertebrate tissues and cells (31). They are derived from a common gene before the differentiation of vertebrates and invertebrates. FABPs are expressed in a variety of tissue cells, including hepatopancreas, muscle, and immune cells, and perform different functions (32). FABPs have immunological functions in invertebrate immune cells (33, 34). Zeng et al. (35) studied the gene expression differences in hemolymph of *Penaeus clarkii* before and after infection with leukoplakia virus by suppression subtractive hybridization (SSH) technology. The authors described that the expression of FABPs was upregulated in hemolymph tissues of *P. clarkii* after infection with infectious hypodermal and hematopoietic necrosis virus (IHHNV). Zhao et al. (36) used the SSH technology to study the expression of different genes in hepatopancreas of susceptible and resistant white spot syndrome virus and found that FABP gene expression was upregulated in hepatopancreas tissue of resistant shrimp. These results suggest that FABPs may have immunological functions. Yang et al. (37) found that the expression level of FABPs in hepatopancreas tissue of IHHNV resistant shrimp was significantly higher than that in susceptible shrimp, suggesting that the gene was involved in inhibiting IHHNV infection in resistant shrimp. Similarly, here we found the highest increased expression of *LvFABP* in hepatopancreas after the shrimp were challenged with VP_AHPND._ This result suggests the involvement of LvFABP in AHPND pathogenesis. Interestingly, the hepatopancreas is also the organ with the most obvious symptoms in AHPND infection, suggesting that the high expression of *LvFABP* in hepatopancreas tissue does not prevent the invasion of pathogenic *Vibrio*, but rather makes the hepatopancreas a target organ for invasion by this pathogen. Expression of *LvFABP* in shrimp at different developmental stages and in different tissues were analyzed using qRT-PCR. Expression of *LvFABP* in the hepatopancreas was significantly higher than that in other organs in the absence of AHPND. Expression of *LvFABP* was also significantly higher at the P23 and P27 developmental stages of *L. vannamei*. Therefore, these developmental stages may be more susceptible, and hepatopancreas tissue is more likely to be the target. The finding that AHPND mainly occurred at the seedling stage of *L. vannamei* is consistent with this result.

Silencing of *Ha*APN1, a Cry1Ac receptor protein in *Helicoverpa armigera*, reportedly decreased the susceptibility of larvae to Cry1Ac toxins (38), while silencing of *HcAPN3*, a gene encoding the Cry1Ab receptor protein was also associated with reduced susceptibility of *Hyphantria cunea* to Cry1Ab (39). In the present study, we likewise investigated the function of LvFABP by dsRNA-mediated silencing. Our results show that ds*LvFABP* significantly silenced *LvFABP* and reduced the mortality of VP_AHPND_ challenged shrimp. Silencing of *LvFABP* also led to reduced numbers of AHPND-causing bacteria in the intestine, stomach, and hepatopancreas of *L. vannamei*. The reason for this reduction is still unclear. One possibility is that the lack of LvFABP might somehow reduce the susceptibility of stomach and hepatopancreas cells to VP_AHPND_ toxins. These results suggest that it is possible to breed AHPND-resistant larvae harboring LvFABP mutation, or to develop a corresponding preparation to block LvFABP receptor sites, as prevention and control measures for shrimp AHPND disease.

Binding specificity between receptor and toxin is also critically important for Cry toxicity. In a previous report, ligand blotting results showed that in *Bombyx mori* and *Hyphandria cunea*, the APN receptors specifically bind to Cry1Aa toxins (40). Similarly, here we used yeast two-hybrid pairwise analysis and GST-pull down and far-western blot assays to determine the interaction between recombinant LvFABP protein and recombinant PirB^vp^ toxin. Recombinant LvFABP directly bound to PirB^vp^ toxin. The yeast two-hybrid pairwise analysis results again demonstrated a specific interaction, suggesting that PirB^vp^ is probably responsible for binding to LvFABP. The role of PirB^vp^ as a ligand for cell surface receptor of shrimp target cells has been recently suggested (39).

LvFABP protein had no antibacterial effect on VP_AHPND_ strain VP-E1 (**Figure 8**). This suggests that LvFABP is not involved in the defense of AHPND through direct bactericidal action, but may mediate the response of AHPND. On the other hand, analysis of the primary structure of LvFABP protein showed that the protein does not have a transmembrane domain and may not be a membrane protein. How and where PirB toxin binds to LvFABP protein in the cell, and what reactions triggered by the binding of the two proteins lead to typical symptoms remain to be studied.

Taken together, our data demonstrate the involvement of LvFABP in AHPND pathogenesis through its action as a VP_AHPND_ toxin interacting protein that mediates hepatopancreas injury. The findings also suggest that LvFABP may be the target protein of PirB^vp^ and may be crucial in the occurrence of disease. We plan to investigate the subcellular localization of LvFABP, specific site of LvFABP binding to toxin, and the signal pathway changes induced by LvFABP binding to toxin. These studies will deepen the understanding of the internal mechanism of LvFABP involved in disease occurrence and could provide new strategies for treatment of AHPND.

## Data Availability Statement

The original contributions presented in the study are included in the article/**Supplementary Material**. Further inquiries can be directed to the corresponding author.

## Ethics Statement

Ethical review and approval was not required for the animal study. The experimental shrimp in the post-larvae stage were cultured in the aquarium of the Institute of Oceanology, Chinese Academy of Sciences and all animal experiments were performed in accordance with accepted standards of humane animal care. No endangered or protected species were used.

## Author Contributions

ML, LW and XG designed the experiment, interpreted the data, and finalized conclusions. XG and ML conducted experimental work and drafted the manuscript. BW and KJ assisted with experiments. All authors read and approved the manuscript.

## Funding

This research was financially supported by National Key R&D Program of China No.2019YFD0900401 and Yellow River Delta Industry Leading Talent Project.

## Conflict of Interest

The authors declare that the research was conducted in the absence of any commercial or financial relationships that could be construed as a potential conflict of interest.

## Publisher’s Note

All claims expressed in this article are solely those of the authors and do not necessarily represent those of their affiliated organizations, or those of the publisher, the editors and the reviewers. Any product that may be evaluated in this article, or claim that may be made by its manufacturer, is not guaranteed or endorsed by the publisher.
