# Supplementary material for: Identification and Expression Analysis of an Interacting Protein (LvFABP) that Mediates Vibrio parahaemolyticus AHPND Toxin Action
Source: Front Immunol. 2022 Jul 4;13:940405. doi: 10.3389/fimmu.2022.940405 (PMC9289683; doi:10.3389/fimmu.2022.940405)
Supplement: Supplementary file 1 [file DataSheet_1.pdf]

Supplementary Material

Supplementary Figures

Supplementary Figure 1. GST-pull down assay of the interaction between LvFABP and PirB^{vp}. M: Protein marker; Lane 1 and Lane 2: LvFABP-GST and PirB^{vp}-His proteins were added to glutathione resin and the mixture was rotated at 4℃ and incubated overnight; Lane 3 and Lane 4: GST and PirB^{vp}-His proteins were added to glutathione resin and the mixture was rotated at 4℃ and incubated overnight; Lane 5 and Lane 6: PBS and PirB^{vp}-His proteins were added to glutathione resin and the mixture was rotated at 4℃ and incubated overnight.

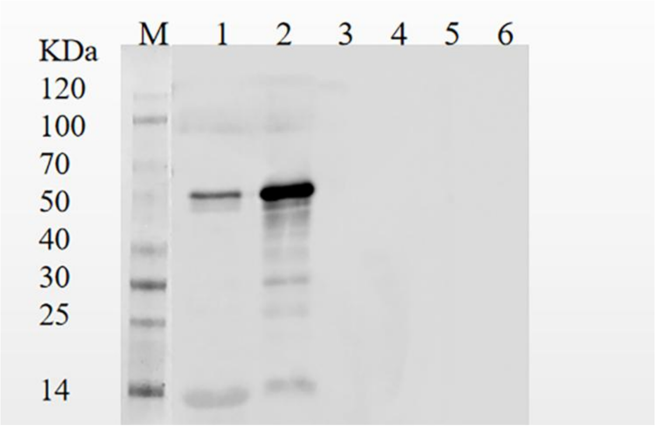

S Fig 1.

Supplementary Figure 2. Far-western blot assay of the interaction between LvFABP and PirB^{vp}. M: Protein marker; Lane 1: Target protein PirB^{vp} toxin; Lane 2: The control group (The mouse serum was used instead of primary antibody).

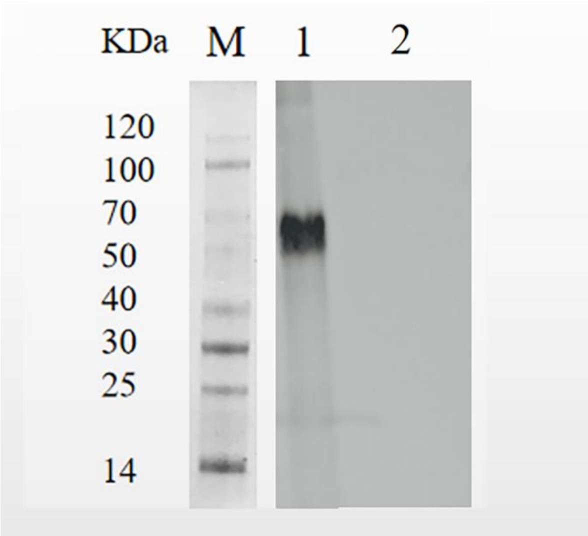

S Fig 2.

Supplementary Figure 3. The silencing efficiency of *LvFABP* RNAi with different dosages of dsRNA. Hepatopancreas tissues were collected after injection different dosages of dsRNA at 48 h. The expression of target genes was detected by qRT-PCR and normalized to the *β-actin* rRNA gene as the internal reference. Each bar represents the mean ± standard deviation (SD) of triplicate experiments. Different letters indicated significantly difference ($P < 0.05$).

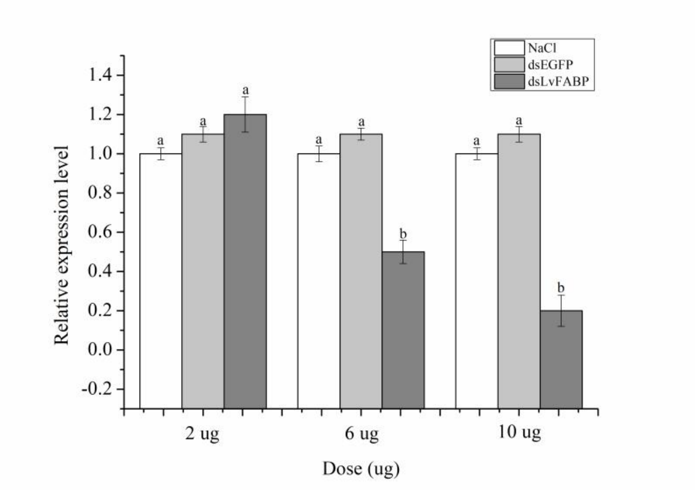

S Fig 3.

Supplementary Figure 4. Relative expression level of *LvFABP* after RNAi in *Litopenaeus vannamei.* Hepatopancreas tissues were collected after RNAi in *Litopenaeus vannamei* at two weeks. The expression of target genes was detected by qRT-PCR and normalized to the *β-actin* rRNA gene as the internal reference. Each bar represents the mean ± standard deviation (SD) of triplicate experiments. Different letters indicated significantly difference ($P < 0.05$).

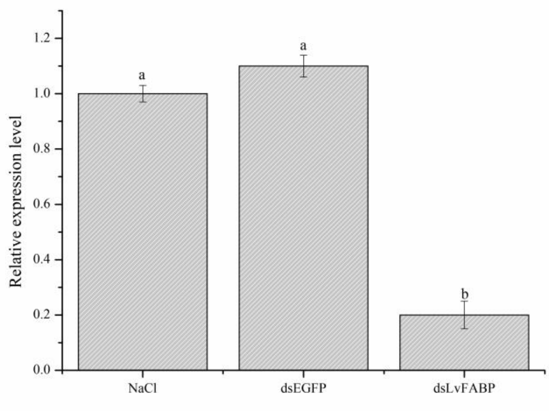

S Fig 4.

Supplementary Figure 5. Reducing of AHPND-causing bacteria in intestine (A), stomach (B), and hepatopancreas (C) of *LvFABP* silenced shrimp. Shrimp intestine, stomach, and hepatopancreas were collected after dsRNA injection (unchallenged) and after infection with VP-E1 at 48 h. Each bar represents the mean ± standard deviation (SD) of triplicate experiments. Different letters indicated significantly difference (P < 0.05).

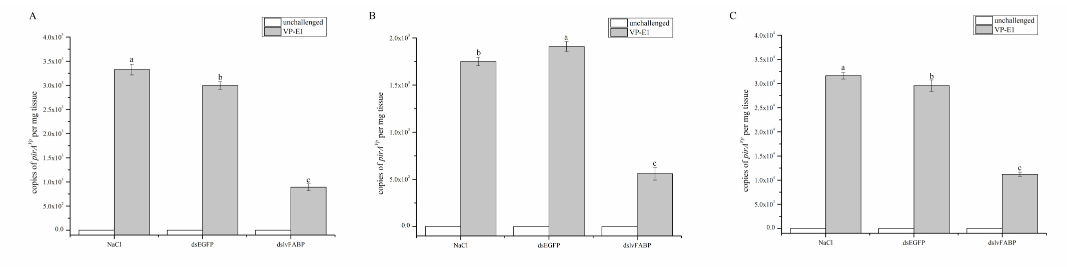

S Fig 5.
